# Distal Left Anterior Cerebral Artery (ACA) Occlusion Masquerading As Middle Cerebral Artery (MCA) Syndrome: To Treat or Not to Treat?

**DOI:** 10.7759/cureus.107412

**Published:** 2026-04-20

**Authors:** Rime Ezzeldin, Alan Hamza, Alzahra'a Al Matairi, Zuhair Ali, Mohamad Ezzeldin

**Affiliations:** 1 Internal Medicine, HCA Florida Ocala Hospital, University of Central Florida College of Medicine, Ocala, USA; 2 College of Medicine, The University of Jordan, Amman, JOR; 3 Clinical Sciences, HCA Houston Healthcare Kingwood, Houston, USA; 4 Clinical Sciences, University of Houston, Houston, USA; 5 Neuroendovascular Surgery, HCA Houston Healthcare Kingwood, Houston, USA

**Keywords:** anterior cerebral artery stroke, ct perfusion imaging, endovascular therapy (evt), mca-mimicking stroke, mechanical thrombectomy (mt)

## Abstract

Anterior cerebral artery (ACA) strokes are rare and often present atypically. We report a case with an isolated left distal A2 segment ACA occlusion presenting with global aphasia and dense right hemiparesis, features typical of a left middle cerebral artery (MCA) syndrome, and representing a severely disabling neurological deficit. CT perfusion imaging confirmed a viable mismatch correlating with the occlusion. The patient underwent a successful thrombectomy with immediate marked neurological improvement and with complete neurological recovery by discharge. While data remain limited, this case illustrates the complex and evolving decision-making process surrounding thrombectomy for distal ACA occlusions with disabling deficits, particularly when perfusion imaging reveals only a 5 cc infarct core and 8 cc of potentially salvageable penumbra, and when recent trial data question population-level benefit. This case highlights the importance of integrating clinical severity with perfusion-based imaging in selecting patients who may still benefit from endovascular intervention despite distal vessel location. Early recognition and intervention in such atypical cases can lead to excellent outcomes.

## Introduction

Anterior cerebral artery (ACA) territory infarctions are uncommon, with the reported incidence of isolated ACA territory infarction ranging from 0.6% to 3% of ischemic stroke [1,2]. Because of robust collateral circulation through the anterior communicating artery and variable vascular anatomy, ACA strokes are frequently underrecognized and may present atypically [3]. Clinically, ACA strokes most commonly present with contralateral lower-extremity-predominant weakness, often accompanied by frontal lobe manifestations such as abulia, apathy, gait disturbance, and urinary incontinence. Language disturbances in ACA infarction are uncommon and, when present, are typically partial or transcortical, related to involvement of the dominant supplementary motor area or anterior cingulate cortex rather than true global aphasia [2-5]. In contrast, middle cerebral artery (MCA) strokes characteristically present with global aphasia, contralateral face and arm weakness, hemisensory loss, and gaze deviation, forming a readily recognizable clinical syndrome [6].

Initial evaluation of suspected ACA stroke relies on non-contrast CT to exclude hemorrhage, followed by CT angiography, although distal ACA occlusions may be missed without careful multiplanar review [3]. Advanced imaging techniques, such as CT perfusion and multiphase CTA, can improve the detection of ACA territory ischemia [7].

Standard acute management of ACA stroke includes intravenous thrombolysis when eligible, while the role of mechanical thrombectomy (MT) remains less clearly defined compared with large-vessel MCA or internal carotid artery (ICA) occlusions according to current stroke guidelines [8]. Despite the rarity of isolated distal ACA occlusions, recent observational data demonstrate that MT in these segments is technically feasible and safe, with high recanalization rates and limited complications [9-13]. However, recent randomized trial data have not demonstrated a clear population-level benefit of thrombectomy for medium- and distal-vessel occlusions, and clinical decisions continue to rely on individual imaging findings and functional deficits [14-16].

Here, we report an isolated distal A2 segment ACA occlusion presenting with an MCA-mimicking clinical syndrome, successfully treated with MT, highlighting the diagnostic and therapeutic challenges posed by atypical ACA strokes.

This article was previously presented as a meeting abstract poster at the 2025 SVIN Annual Meeting on November 19-22, 2025

## Case presentation

**Figure 1:**
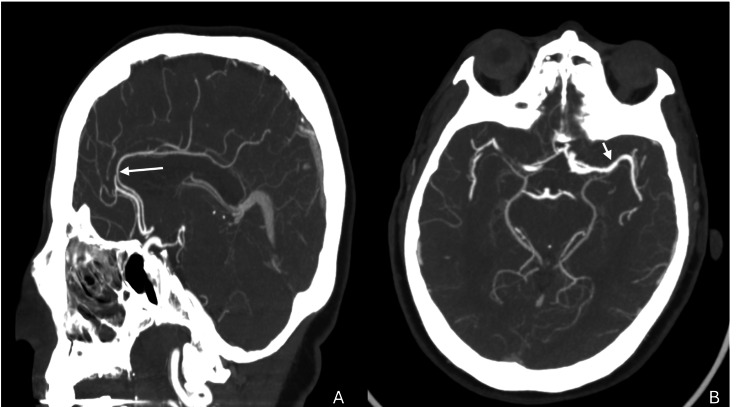
CT angiography (CTA) demonstrating distal left anterior cerebral artery occlusion. (A) Sagittal view demonstrating an occlusive thrombus in the distal left A2 segment (arrow). (B) Axial view demonstrating preserved patency of the left middle cerebral artery (arrow).

**Figure 2:**
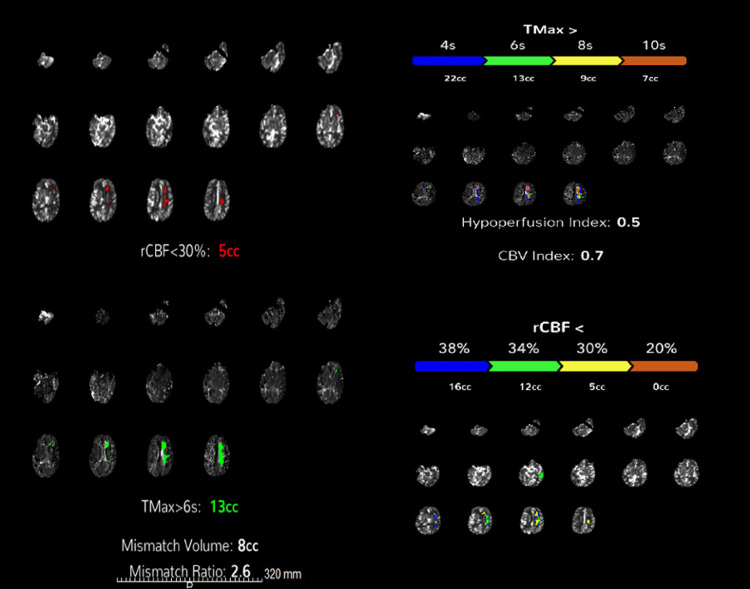
CT perfusion (CTP) imaging demonstrating ischemic core and penumbra. Multimodal CTP maps demonstrate perfusion abnormalities in the left ACA territory. The relative cerebral blood flow (rCBF <30%) map shows a small infarct core of 5 cc. The Tmax >6s map reveals a larger area of hypoperfusion measuring 13 cc, resulting in a mismatch volume of 8 cc and a mismatch ratio of 2.6. The hypoperfusion index is 0.5, and the CBV index is 0.7, suggesting relatively preserved collateral status and a favorable perfusion profile. ACA: anterior cerebral artery; CBV: cerebral blood volume

A 69-year-old woman with a history of hypertension and remote breast cancer in remission was airlifted to the emergency department with sudden-onset global aphasia, right-sided hemiparesis/hemisensory loss, and gaze deviation. Her National Institutes of Health Stroke Scale (NIHSS) score was 18, indicating moderate-to-severe ischemic stroke, clinically resembling a left MCA syndrome [17]. She was fully independent at baseline, a non-smoker, and not on anticoagulation. Non-contrast CT of the head showed no acute findings. CTA revealed a distal left A2 segment ACA occlusion and a patent MCA (Figure 1). CT perfusion demonstrated a 5 mL infarct core and 8 mL of ischemic penumbra, corresponding to a mismatch ratio of 2.6 in the left ACA territory (Figure 2).

After confirming the absence of active malignancy or brain metastases, intravenous tenecteplase was administered. Informed consent was obtained from the family after a detailed discussion addressing the severity of the patient’s neurological deficits, the atypical presentation, and limited evidence supporting MT for distal ACA occlusions in the context of recent neutral trials. The patient underwent emergent cerebral angiography under general endotracheal anesthesia with the intent to proceed with thrombectomy.

**Figure 3:**
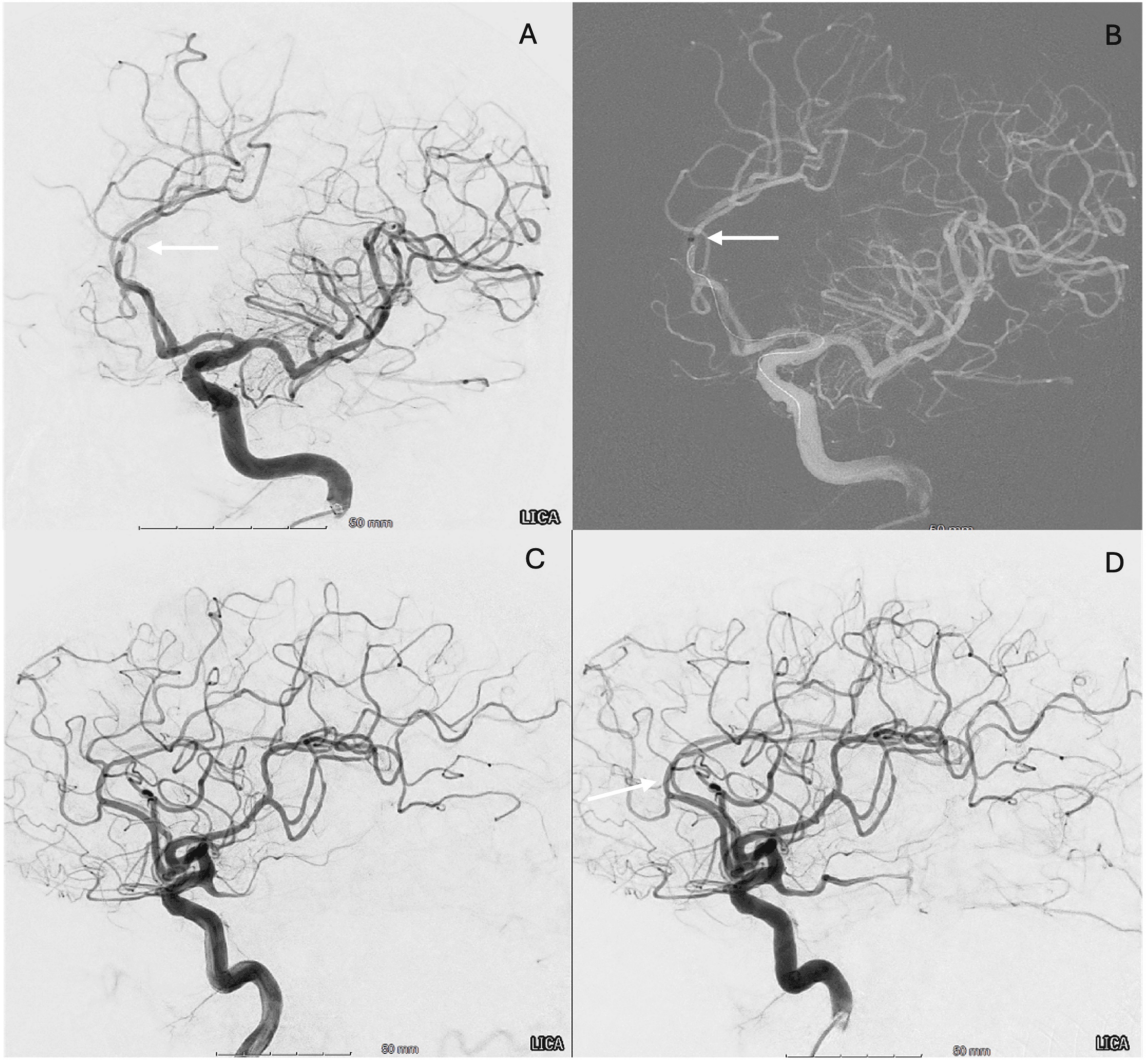
Cerebral angiography demonstrating distal anterior cerebral artery (ACA) thrombectomy and complete reperfusion. (A) Left internal carotid artery (ICA) oblique angiogram demonstrating a partially occlusive thrombus in the distal left A2 segment of the ACA (arrow), with preserved antegrade flow distal to the thrombus. (B) Roadmap image demonstrating the tip of the Zoom 35 aspiration catheter positioned in contact with the thrombus (arrow). (C, D) Post-thrombectomy left ICA angiograms demonstrating complete reperfusion of the left ACA territory, consistent with Thrombolysis in Cerebral Infarction (TICI) grade 3 recanalization (arrow).

Right common femoral artery access was established, and an 8 French (8F) sheath was placed. Due to a type III aortic arch and tortuous left common carotid artery (CCA), multiple catheter systems were attempted without successful engagement of the left CCA. Ultimately, a Zoom 88 support catheter (Imperative Care, Campbell, CA, USA) was advanced over a 6F VTK into the left CCA. Selective left ICA injection revealed normal MCA filling, mild diffuse intracranial atherosclerosis, and a partially occlusive thrombus in the distal left ACA. A Zoom 35 reperfusion catheter (Imperative Care, Campbell, CA, USA) was advanced over an 0.018-inch Aristotle microwire (Scientia Vascular, West Valley City, UT, USA) to the thrombus face in the left distal A2-A3 segment. Contact aspiration was performed for one minute using an aspiration pump, and a light-red thrombus was retrieved. Post-aspiration angiography showed complete Thrombolysis in Cerebral Infarction (TICI) grade 3 recanalization (Figure 3), with a puncture-to-recanalization time of 32 minutes, due to the previously described challenging anatomy. The procedure was completed without complications. The patient demonstrated immediate and marked neurological improvement upon emergence from anesthesia, with the NIHSS improving from 18 on admission to 4 post-procedure. She regained the ability to speak and follow commands and was subsequently transferred to the Neuro ICU in stable condition.

**Figure 4:**
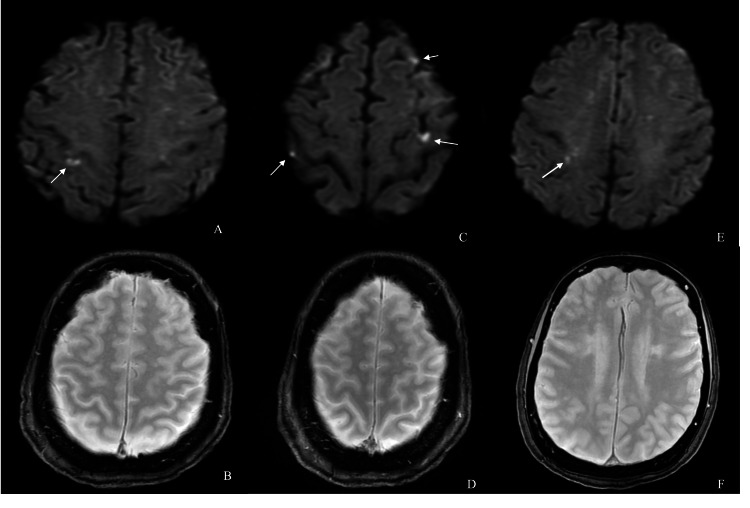
Post-procedural MRI demonstrating multifocal ischemic lesions without hemorrhagic transformation. (A, C, E) Axial diffusion-weighted imaging (DWI) sequences demonstrate multiple small and subtle, scattered areas of diffusion restriction in the periventricular and subcortical regions of both cerebral hemispheres (arrows), consistent with recent ischemic infarcts. (B, D, F) Axial gradient echo (GRE) sequence shows no evidence of hemorrhagic transformation.

Post-procedure MRI brain demonstrated several recent periventricular and subcortical ischemic lesions in both cerebral hemispheres without evidence of hemorrhage or large territorial infarction (Figure 4). Cardiology was consulted for a comprehensive cardiac evaluation. Electrocardiogram demonstrated sinus tachycardia, and echocardiography showed normal left ventricular function with no intracardiac shunt on bubble study. Inpatient telemetry demonstrated a normal sinus rhythm throughout hospitalization, and outpatient 30-day cardiac monitoring was recommended to evaluate for occult atrial fibrillation or flutter, which was negative. CT abdomen and pelvis revealed a renal lesion, and oncology follow-up was planned due to cancer history. The patient continued to improve clinically. At discharge, the NIHSS was 0, and the functional outcome was excellent with a modified Rankin Scale (mRS) score of 1. She was discharged on dual antiplatelet therapy followed by lifelong aspirin monotherapy.

## Discussion

This case highlights an exceptionally rare presentation of acute ischemic stroke in which an isolated distal A2 ACA occlusion produced a clinical syndrome indistinguishable from a proximal MCA stroke. Although ACA infarctions may cause language disturbances when the dominant supplementary motor area or anterior cingulate cortex is involved, published cohort studies and systematic reviews consistently describe ACA strokes as clinically distinct from MCA syndromes, with predominant lower extremity weakness and frontal behavioral features. Reports of ACA-related aphasia describe partial or atypical language deficits rather than global aphasia accompanied by dense hemiparesis, underscoring the unique clinical presentation observed in this case [1-5].

The precise anatomical basis for the global aphasia observed in this patient cannot be definitively established, as no clear vascular variant was identified on CTA or angiography. However, infarction within the dominant ACA territory, particularly involving the medial frontal lobe, including the supplementary motor area, anterior cingulate cortex, and adjacent deep frontal white matter, has been associated with severe language dysfunction that can clinically mimic global aphasia. Disruption of interhemispheric callosal connections and frontal-subcortical language networks may further contribute to this presentation. Similarly, the presence of gaze deviation, which initially suggested an MCA syndrome, can be explained despite preserved MCA patency, as ischemia affecting medial frontal regions involved in motor planning and eye movement control may produce findings typically attributed to frontal eye field involvement. Together, these mechanisms likely explain how an isolated distal ACA occlusion in this patient resulted in neurological deficits closely resembling a proximal MCA stroke [2-5].

In this case, imaging and clinical mismatch were key in the diagnosis. While conventional imaging may not immediately reveal distal ACA involvement, perfusion studies and clinical-anatomic correlation can identify the true site of occlusion. Techniques such as CTA with sagittal maximum intensity projection (MIP) reconstructions and CT perfusion can clarify perfusion asymmetries and help localize occlusions that might otherwise be mistaken for MCA syndromes [7]. CTA confirmed preserved MCA flow, and CT perfusion demonstrated a mismatch ratio >2.5 with a small infarct core, raising questions about the strength of the indication for MT in this context.

Regarding treatment, this case adds to the growing body of literature supporting MT for distal ACA occlusions, including the A3 segment and callosomarginal or pericallosal branches, with recanalization rates exceeding 80% and minimal complications [7-12]. Device-level feasibility has been demonstrated using both stent retrievers and aspiration catheters, with successful deployment even through long, curved, and narrow ACA paths in the A2-A4 segments [7,12].

Recent randomized trials evaluating MT for medium- and distal-vessel occlusions, including ESCAPE-MeVO [14], DISCOUNT [15], and DISTAL [16], have provided important yet controversial insights. Across these trials, thrombectomy strategies have been heterogeneous and operator-dependent, incorporating stent retrievers, aspiration catheters, or combined approaches, reflecting real-world practice rather than standardized procedural protocols. Notably, distal ACA occlusions involving the A2 segment were explicitly included in all three trials, directly situating the present case within the contemporary MeVO/DVO evidence base. In addition, there is no universally accepted definition of MeVO/DVO; definitions vary across trials: DISCOUNT classifies ACA occlusions as distal, ESCAPE-MeVO classifies A2-A3 ACA occlusions as MeVO, and DISTAL includes A1-A3 ACA within a combined "medium or distal" category, further complicating cross-trial comparisons and generalizability [14-16].

Endovascular therapy for MeVO/DVO in the trials demonstrated technical feasibility and acceptable safety profiles; however, none showed a statistically significant functional benefit over best medical therapy at the population level. In particular, the DISTAL randomized trial showed no significant difference in 90-day functional outcomes or mortality between endovascular therapy and best medical treatment alone for isolated MeVO/DVO strokes, including A2 and A3 ACA occlusions, despite acceptable safety and recanalization rates [16]. Earlier registry data, such as EVATRISP, show comparable safety and outcome profiles between IV thrombolysis and MT in ACA strokes, regardless of whether the occlusion is proximal (A1) or distal (A2-A3) [10].

More recent late-breaking data presented at the International Stroke Conference 2026 further illustrate the rapidly evolving evidence base for thrombectomy in medium- and distal-vessel occlusions. Emerging results from the ORIENTAL-MeVO randomized trial suggest that endovascular thrombectomy (EVT) may improve functional outcomes in selected patients with medium-vessel occlusions, particularly those presenting with moderate-to-severe neurological deficits. In parallel, the DISTALS trial evaluating a low-profile stent retriever designed for distal vessels demonstrated high rates of successful reperfusion with favorable safety outcomes. Although these studies were presented at conferences and have not yet been fully published, they suggest that advances in device technology and improved patient selection may influence the role of thrombectomy in distal occlusions [18,19].

Taken together, these mixed and evolving results may simultaneously discourage intervention while also prompting some clinicians to pursue distal occlusions in highly selected patients. Importantly, the 2026 AHA/ASA acute ischemic stroke guideline assigns a Class of Recommendation III (No Benefit) to EVT for medium and distal vessel occlusions, including distal ACA occlusions, while simultaneously emphasizing that further research is needed to determine whether faster workflows, aspiration-first strategies, intra-arterial thrombolysis, or newer thrombectomy devices, particularly in patients presenting with more substantial neurological deficits, may clarify a potential role for EVT in this population [8]. This uncertainty underscores the critical importance of careful patient selection based on clinical severity, disabling deficits, and favorable imaging profiles rather than vessel caliber alone. The favorable outcome observed in this patient with a distal A2 ACA occlusion and MCA-mimicking presentation illustrates the potential value of this patient-centered approach.

## Conclusions

Distal ACA occlusions can present with profoundly disabling, MCA-mimicking clinical syndromes and may be easily overlooked during initial evaluation. This case illustrates that, despite largely neutral early randomized trials of MT for medium- and distal-vessel occlusions, selected patients may still benefit from endovascular intervention when guided by individualized, imaging-based decision-making beyond traditional large-vessel paradigms. In carefully chosen cases, such an approach may result in rapid and substantial neurological recovery.

## References

[1] Cho H, Kim T, Kim YD (2022). A clinical study of 288 patients with anterior cerebral artery infarction. J Neurol.

[2] Moncayo-Gaete J, Bogousslavsky J (2025). Anterior cerebral artery stroke syndromes. MedLink Neurology.

[3] Matos Casano HA, Tadi P, Ciofoaia GA (2023). Anterior cerebral artery stroke. StatPearls [Internet].

[4] Kim DJ, Marzoughi S, Field TS (2022). Teaching NeuroImage: atypical anterior cerebral artery syndrome from pericallosal artery infarct. Neurology.

[5] Rubens AB (1975). Aphasia with infarction in the territory of the anterior cerebral artery. Cortex.

[6] Nogles TE, Galuska MA (2023). Middle cerebral artery stroke. StatPearls [Internet].

[7] Goyal M, Cimflova P, Ospel JM, Chapot R (2021). Endovascular treatment of anterior cerebral artery occlusions. J Neurointerv Surg.

[8] Prabhakaran S, Gonzalez NR, Zachrison KS (2026). 2026 guideline for the early management of patients with acute ischemic stroke: a guideline from the American Heart Association/American Stroke Association. Stroke.

[9] Chung GH, Kwak HS, Park JS, Lee JM (2017). Manual aspiration thrombectomy with a Penumbra catheter for acute anterior cerebral artery occlusion. Interv Neuroradiol.

[10] Filioglo A, Simaan N, Honig A (2022). Outcomes after reperfusion therapies in patients with ACA stroke: a multicenter cohort study from the EVATRISP collaboration. J Neurol Sci.

[11] Pfaff J, Herweh C, Pham M, Schieber S, Ringleb PA, Bendszus M, Möhlenbruch M (2016). Mechanical thrombectomy of distal occlusions in the anterior cerebral artery: recanalization rates, periprocedural complications, and clinical outcome. AJNR Am J Neuroradiol.

[12] Miszczuk M, Kleine JF, Riegler C, Bauknecht HC, Bohner G, Siebert E (2022). Mechanical thrombectomy of acute occlusions of individual distal anterior cerebral artery branches. J Clin Neurosci.

[13] Dabhi N, Mastorakos P, Sokolowski J, Kellogg RT, Park MS (2022). Mechanical thrombectomy for the treatment of anterior cerebral artery occlusion: a systematic review of the literature. AJNR Am J Neuroradiol.

[14] Goyal M, Ospel JM, Ganesh A (2025). Endovascular treatment of stroke due to medium-vessel occlusion. N Engl J Med.

[15] Clarençon F, Durand-Zaleski I, Premat K (2024). Evaluation of mechanical thrombectomy in acute ischemic stroke related to a distal arterial occlusion: a randomized controlled trial. Int J Stroke.

[16] Psychogios M, Brehm A, Ribo M (2025). Endovascular treatment for stroke due to occlusion of medium or distal vessels. N Engl J Med.

[17] Brott T, Adams HP Jr, Olinger CP (1989). Measurements of acute cerebral infarction: a clinical examination scale. Stroke.

[18] DISTALS Investigators (2026). Distal Ischemic Stroke Treatment With Adjustable Low-profile Stentriever (DISTALS). Presented at the International Stroke Conference 2026.

[19] ORIENTAL-MeVO Investigators (2026). Evaluation of Endovascular Treatment in Acute Intracranial Distal Medium Vessel Occlusion Stroke. International Stroke Conference 2026.

